# Interventions after Arterial Switch: A Single Low Case-Volume Center Experience

**DOI:** 10.3390/medicina57050401

**Published:** 2021-04-21

**Authors:** Karolis Jonas, Virginijus Jakutis, Rita Sudikienė, Virgilijus Lebetkevičius, Virgilijus Tarutis

**Affiliations:** 1 Center of Cardiothoracic Surgery, Clinic of Cardiovascular Diseases, Institute of Clinical Medicine, Vilnius University Faculty of Medicine, Santariskiu St. 2, LT-08661 Vilnius, Lithuania; rita.sudikiene@santa.lt (R.S.); virgilijus.lebetkevicius@santa.lt (V.L.); virgilijus.tarutis@santa.lt (V.T.); 2 Clinic of Anesthesiology and Intensive Care, Institute of Clinical Medicine, Vilnius University Faculty of Medicine, Santariskiu St. 2, LT-08661 Vilnius, Lithuania; virginijus.jakutis@santa.lt

**Keywords:** late complications after arterial switch operation, pulmonary stenosis after arterial switch operation, aortic arch obstruction after arterial switch operation, catheter-based treatment after arterial switch operation

## Abstract

*Background and Objectives*: With the growing population of arterial switch operation survivors, the rate of late complications associated with the operation is growing as well. The aim of this publication is to share our experience and encourage collaboration between congenital cardiac surgeons and interventional cardiologists in treating late complications after arterial switch operation. *Materials and Methods*: A retrospective analysis of Vilnius University Santaros Clinics Cardiothoracic Surgery Centre arterial switch operation survivors who underwent additional treatment for late neo-pulmonary artery stenosis and aortic arch obstruction between 1989 and 2019 was conducted. *Results*: Out of 95 arterial switch operation survivors 14 (15%) underwent 36 reinterventions. The majority were treated for neo-pulmonary stenosis. The median time from arterial switch operation to the first reintervention was 1.4 years (interquartile range, 2 months to 2.4 years). 1, 3, 5, and 10 years intervention-free survival in patients treated for neo-pulmonary stenosis and aortic arch obstruction was 98, 94, 94, and 93% vs. 95, 94, 94, and 93%, respectively. There were no complications associated with redo surgical procedures, while eight patients who underwent catheter-based interventional treatment had treatment-related complications, including one death. *Conclusions*: Both neo-pulmonary stenosis and aortic arch obstruction (new aortic coarctations or aortic recoarctations) tend to develop in the first decade after an arterial switch operation. Surgical and catheter-based interventional treatment with good results is possible even in a small volume center. Close collaboration of the congenital heart team (congenital cardiac surgeons and interventional cardiologists) in choosing the best treatment option for an individual patient helps to minimize the risk of potential complications.

## 1. Introduction

Transposition of the great arteries is a congenital malformation of the heart in which a discordant ventriculoarterial connection develops during the embryogenesis. The prevalence of this malformation among patients with congenital heart defects is 5–7% [1]. The only definitive treatment option is surgery. The gold standard is the arterial switch operation as described by Jatene with LeCompte maneuver [2,3]. Arterial switch operation, when performed in high-expertise high case-volume centers, has an extremely low in-hospital mortality rate. Increasing amount of small and medium centers reports satisfactory in-hospital mortality rate [4,5]. Since 1975, when the first successful arterial switch was performed, the population of these patients is growing. The arterial switch operation requires cutting and stitching back the major vessels, transferring the ostia of coronary arteries to the neo-aortic root, and performing the LeCompte maneuver—translocation of the neo-pulmonary artery in front and on top of the neo-aorta, which can lead to tension in the main neo-pulmonary artery and its branches. In some cases, there is a necessity to repair aortic coarctation or hypoplastic aortic arch. Up to 1/3 arterial switch patients may develop obstructive lesions in the repaired areas and might need an additional intervention in the late post-operative period [6,7,8,9]. In this article, we describe our experience in treating late complications after arterial switch operation.

## 2. Materials and Methods

A retrospective analysis of patients who underwent arterial switch for transposition of the great arteries from 1989 to 2019 at Vilnius University Hospital Cardiothoracic Surgery Centre was conducted. The study was approved by a local ethics committee.

An initial analysis of all patients’ medical records was performed. Patients who underwent a surgical or interventional procedure to repair a right-sided (right ventricle outflow tract obstruction, valvular and supravalvular stenosis of the neo-pulmonary artery or its branches), or left-sided lesion (left ventricle outflow tract obstruction, valvular or supravalvular aortic stenosis, primary aortic coarctation or re-coarctation of the aorta) were further analyzed. The patients were stratified in to two groups according the first reintervention location. Data regarding these patients’ age, original diagnosis, surgical history, redo (both surgical and interventional) procedure count and type, time from arterial switch to redo procedure, indications for a redo procedure, redo procedure complications and their management, and patient outcomes were gathered from patients medical records.

### 2.1. Statistical Analysis

Numerical variables are presented as medians and interquartile range (IQR) or range where applicable, as all numerical data was not distributed normally. All categorical variables are presented as counts and percentages. Two-sample Wilcoxon test was used to compare numerical variables between the two groups. Fisher Exact Test was used to compare categorical variables between the two groups. As the main endpoint of this study is at least one reintervention after the arterial switch, reinterventions free survival was estimated using the Kaplan–Meier method. A log-rank test was used to compare survival estimates between the two groups. A *p*-value of less than 0.05 was chosen to represent statistical significance. Statistical analysis was performed using R statistical software package version 3.4.4 (R Core Team, R Foundation for Statistical Computing: Vienna, Austria, 2018) [10].

### 2.2. Operative Technique, Follow-Up Procedure and Management of Late Arterial Switch Complications

All patients underwent a standard arterial switch operation with the LeCompte manoeuver. Coronary button reimplantation to the neo-aortic root was done using slit incisions in the Valsalva sinuses walls or by utilizing the trap-door technique. Reconstruction of the neo-pulmonary root was done using a single pantaloons shaped untreated autologous pericardium patch. If present, ventricular septal defect was closed through the right atriotomy using a synthetic polyester patch. Primary aortic coarctation was repaired by resecting the coarctation and anastomosing the aortic arch and the descending aorta in an end to end fashion. If the coarctation was associated with hypoplastic aortic arch, the coarctation was resected, the arch was incised longitudinally, a direct anastomosis between the back wall of the aorta and the aortic arch was performed, and the front wall was enlarged using untreated autologous pericardial patch or decellularised bovine or equine pericardial patch. After the operation, the patients were treated in a dedicated pediatric intensive cardiac care unit, where they were managed by dedicated intensive care physicians and nurses. In the ward, patients were managed by a dedicated team of pediatric cardiologists and congenital cardiac surgeons. After discharge, patients were followed by the same team of pediatric cardiologists in an out-patient clinic. The routine follow-up procedure in our center is as follows: the first out-patient visit is held 1 week after discharge, then after 2 weeks, 1 month, 3 months, 6 months, and finally once every year. A routine check-up includes full clinical exam, electrocardiogram and an echocardiographic study. Additional investigations are appointed as needed. If late arterial switch complications are present, each patient’s situation is discussed in a multidisciplinary team meeting held every week. The multidisciplinary team includes congenital cardiac surgeons, interventional cardiologists specializing in congenital cardiac interventions, pediatric cardiologists, cardiac anesthetists and intensive care physicians. The treatment option (surgery vs. intervention) is individually selected for the individual patient during the meeting.

## 3. Results

During the study period, 124 patients underwent an arterial switch operation at Vilnius University Santaros Clinics Cardiothoracic Surgery Centre. The median case-load was 4 operations per year (IQR 1 to 6 operations per year). The early post-operative mortality rate was 23%. The main cause, of high early-mortality rates were poor results in the beginning of the arterial switch program. In the first decade, 24 patients were operated, only six survived (25%). In the second decade, 56 patients were operated, 47 survived (82%). In the last decade, 44 patients were operated, and 42 survived (95%). Poor results in the first decade can be attributed to early learning period. However, one has to keep in mind, that Lithuania regained independence in 1990. During the first decade of a newly established country posed economic and political difficulties, which also influenced the health care system. As political and economic situation improved, health care system improved as well. As our team advanced the learning curve, the early survival rates improved. The first, second, and third decade survival rates were 25%, 82% and 95% respectively (*p*-value < 0.05). During the post-operative period, there were no patients with residual ventricular septal defects among the survivors. None of our surviving patients had any coronary related events. No patients required treatment for neo-aortic root enlargement or neo-aortic valve regurgitation.

Out of 95 survivors, 14 patients underwent a total of 36 additional catheter-based interventional and surgical treatment procedures for neo-pulmonary artery stenosis and aortic arch obstruction. Seven patients underwent treatment for neo-pulmonary stenosis as a first reintervention. They were allocated to the “neo-pulmonary stenosis group”. None of these patients developed left-sided lesions during the follow-up period. The remaining 7 patients underwent treatment for aortic arch obstruction as the first reintervention. These patients were allocated to the “aortic arch obstruction group”. Two of them were treated for newly developed aortic coarctation and five patients were treated for aortic recoarctation. Out of the latter two patients developed neo-pulmonary artery stenosis.

The demographic data summary, including female to male ratio, transposition of the great arteries form, coronary artery pattern, and additional cardiovascular malformations, is shown in Table 1. The female to male ratio in both groups was similar. There were more patients in the aortic arch obstruction group who underwent arterial switch operation due to the Taussig-Bing transposition. However, this difference was not statistically significant (Fisher Exact Test *p*-value is 0.28). There were more patients who had aortic coarctation repaired during arterial switch operation in the aortic arch obstruction group (Fisher Exact Test *p*-value is equal to 0.1). There were significantly more patients who required hypoplastic aortic arch repair during arterial switch operation in the aortic arch obstruction group (Fisher Exact Test *p*-value is less than 0.05).

**Figure 1:**
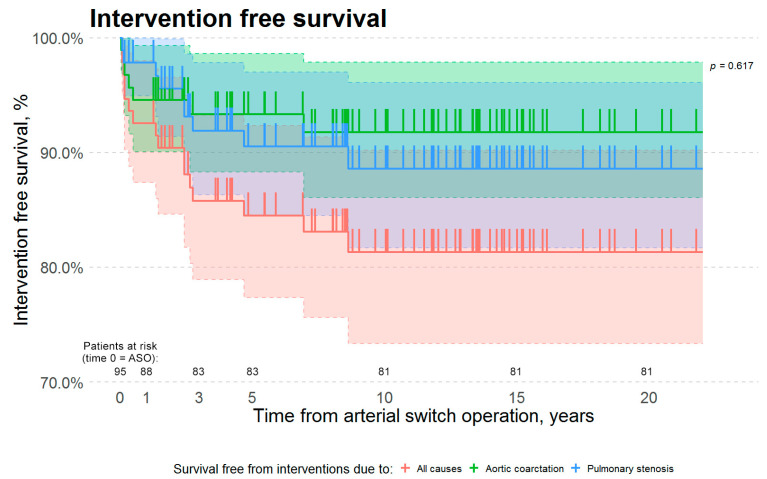
Kaplan–Meier survival estimates. The red curve represents survival free from interventions due to all causes. The green curve represents survival free from interventions due to aortic arch obstruction. The blue curve represents survival free from interventions due to neo-pulmonary artery stenosis. There is no statistically significant difference in survival free from interventions due to aortic arch obstruction vs. neo-pulmonary artery stenosis (Kaplan–Meier Log-Rank *p*-value is 0.617). *Y*-axis scale was altered to a range from 70% to 100%. Abbreviations: ASO—arterial switch operation.

The median time from arterial switch to first reintervention due to any cause was 1.4 years (IQR 2 months to 2.4 years). In our cohort, patients required first reintervention for aortic re-coarctation earlier, than patients who required the first reintervention for neo-pulmonary artery stenosis. Median times from arterial switch to the first reintervention for aortic re-coarctation and for neo-pulmonary artery stenosis were 4 months (IQR 2.3 months–1.6 years) and 2.4 years (IQR 1.4 years–2.8 years), respectively (Table 2). However, this difference was not statistically significant (two-sample Wilcoxon W = 25, *p*-value is greater than 0.05). The 1, 3, 5 and 10 years survival free from all interventions was 93, 88, 88, and 85%, respectively (Figure 1), while 1, 3, 5 and 10 years survival free from interventions due to neo-pulmonary artery stenosis was 98, 94, 94, and 92%, respectively, and due to aortic re-coarctation was 95, 94, 94, and 93%, respectively. The data suggest an earlier onset of left-sided obstruction. However, due to a low number of these late complications, there was no statistical significance (Kaplan–Meier survival curve log-rank *p*-value is greater than 0.05). In our cohort, both neo-pulmonary artery stenosis and aortic re-coarctation tend to develop in the first decade after the arterial switch operation (Figure 1).

Three of our patients underwent only one reintervention (two surgical and one catheter-based interventional treatment, and the remainder 11 patients underwent more than one reintervention. The median number of procedures performed to our patients was 2 in both groups (Table 2). Out of the 11 patients who underwent more than one repeat procedure, only one patient had surgical repair as the first reintervention. Three patients underwent catheter-based interventional treatment followed by surgical treatment. One of them required additional treatment after the last surgery. The most common treatment option in our cohort was balloon angioplasty both in patients with neo-pulmonary artery stenosis and aortic arch obstruction. However, patients with neo-pulmonary artery stenosis underwent surgical repair more frequently (Fisher Exact Test *p*-value is less than 0.05). Only two stenting procedures were performed. One patient underwent stenting for stenosis of the right neo-pulmonary artery branch, and the other patient underwent stenting for repeat aortic recoarctation.

None of the surgically treated patients had complications related to redo cardiac surgery (Table 2). In eight patients who underwent catheter-based interventional treatment there were ten complications related to the procedures. Two patients underwent diagnostic catheterization procedure instead of planned catheter-based interventional treatment. In the first patient, a balloon catheter could not be guided across the stenosis in the right neo-pulmonary artery. This patient later underwent surgical repair. In the second patient, a planned stenting of repeat aortic re-coarctation was terminated, because a required sized introducer (10 Fr) did not fit in the access artery (the patient had three previous balloon angioplasty procedures performed through the same artery). On post-procedure day 2, this patient developed a thrombosis of the right femoral artery, which was used as the access site. As the distal circulation was adequate, the patient was treated with low molecular weight heparin. The patient underwent successful stenting of the aortic recoarctation 2.7 years later. During the procedure, an angiogram of the lower extremities was performed. An occlusion of the superficial femoral artery used as an access site during the previous procedure was present. However, good collateral flow in the distal leg was also visible. Other complications in our cohort include four cases of localized target site dissection. In three cases the complication resolved without treatment, and in one case it was managed by additional balloon angioplasty using a smaller balloon with lower pressure. One patient had acute bleeding from the access site artery, which required immediate surgical repair. Another patient developed access site artery thrombosis on post-procedure day 3, which resolved completely in 5 days with adequate anticoagulation using low molecular weight heparin. Catheter-based interventional treatment complications related to the access site vessel seem to be more frequent in patients treated for late aortic arch obstruction. However, this difference lacks statistical significance (Fisher Exact Test *p*-value is 0.2).

There was one death in our cohort related to catheter-based interventional treatment procedure. Two and a half years after the arterial switch operation this patient developed an acute infection, which led to the development of septic right hip and knee joints arthritis. The patient was treated in an outside hospital. After a month of antibiotics and non-steroid anti-inflammatory medicine the patient’s condition improved. Upon discharge, the patient developed acute dyspnea, cyanosis and heart failure. The patient was transferred to our intensive cardiac care unit with a suspected right heart obstruction. The echocardiographic exam showed an enlarged and barely contracting right ventricle, though there were no signs of main neo-pulmonary artery stenosis. After multidisciplinary team discussion a decision to perform a right heart catheterization was made, and the patient underwent an urgent procedure. A right femoral vein was selected as an access site. Immediately after placing an introducer, the patient’s heart arrested. Subsequent angiography under cardiac resuscitation showed no blood flow through the neo-pulmonary artery and evidence of thrombi in the right femoral and iliac veins. Acute intravenous thrombolysis was started, and cardiopulmonary resuscitation efforts were maintained for 2 h without success.

However, all of these complications are not specific to patients after arterial switch operation and can be seen during or after any cardiac catheterization procedure.

## 4. Discussion

Late complications related with the arterial switch operation can be divided into two main categories: the lesions of the right side and all other, the former being more common. There are reports of series where more than 50% of patients develop neo-pulmonary artery stenosis [7,12,13,14,15]. This is also true in the setting of low case volume as illustrated by our experience. Out of 124 arterial switch operations performed at our center in more than three decades, 95 (77%) patients survived. Out of the survivors, 14 (15%) patients’ had developed late complications requiring treatment. Seven (50%) patients were treated for neo-pulmonary stenosis, five (36%) were treated for aortic arch obstruction (including both new onset aortic coarctation and aortic recoarctation), and two (14%) were treated for both aortic arch obstruction and neo-pulmonary artery stenosis.

There are a lot of discussions on the cause of late neo-pulmonary artery stenosis after arterial switch operation. The nature of the operation itself involves transecting both major arteries and suturing them back into the correct place. The neo-aorta is formed by suturing the distal ascending aorta to the pulmonary root arising from the left ventricle. The neo-pulmonary artery is formed by suturing the distal pulmonary artery with the aortic root arising from the right ventricle [2]. The operation includes the transfer of the coronary ostia. The coronary ostia are excised with a rim of the aortic wall and then reimplanted to the neo-aortic root. The technique of the reimplantation depends on the coronary anatomy and the preference of the surgeon. The problem is that excising coronary ostia buttons from the aortic root leaves defects which need to be reconstructed. There are a lot of techniques for harvesting coronary buttons and reconstructing the neo-pulmonary root afterwards. Some surgeons excise the coronary buttons with almost all surrounding tissue of the Valsalva sinus, while others harvest with only a small rim of tissue for later suturing. In some centers, surgeons excise coronary buttons while leaving the distal border of the aortic root intact, which they later use for direct anastomosis with the distal pulmonary artery. Different materials are used for reconstruction of the vessel wall including synthetic patch material, tissue engineering produced decellularised patch material, untreated or fixed autologous pericardium in the form of a single pantaloons shaped patch or as two separate patches [2,3,6,7,15,16,17,18,19,20,21]. There is no unanimous agreement of which technique contributes more to neo-pulmonary artery stenosis. Some authors argue that it is better to perform a direct neo-pulmonary root and distal pulmonary artery anastomosis. In their opinion, foreign material (both autologous and synthetic) is inferior to vascular tissue in regards to the growth of the anastomotic site [6,7,16,17,18,19,21]. However, there are patients with neo-pulmonary stenosis in their groups as well. Other authors state the opposite—in their experience, neo-pulmonary artery stenosis is associated with direct anastomosis between the aortic root and distal pulmonary artery [15]. All of our patients’ neo-pulmonary roots were reconstructed using a single pantaloons shape untreated autologous pericardium patch. Another operative technique factor that could potentially be the cause of the supravalvular neo-pulmonary artery stenosis is the LeCompte maneuver. In 1981, LeCompte purposed to translocate the pulmonary bifurcation on top of the aorta during the arterial switch operation. This manoeuver not only untangles the transposed major vessels, eases the anastomosis of both neo-aorta and neo-pulmonary valve, but also reduces the possibility of external compression on the translocated coronary arteries [3]. The manoeuver, however, straddles the pulmonary bifurcation across the aorta. In turn, this may put excessive tension on the pulmonary artery and its branches, which can be the cause of the supravalvular neo-pulmonary stenosis [9,22]. Furthermore, inadequate dissection of the neo-pulmonary root ant pulmonary artery branches can lead to impaired growth of the neo-pulmonary aorta and be the cause of the neo-pulmonary artery stenosis, though extensive dissection of the pulmonary artery branches can be the cause of excessive adhesion formation, which may also be the cause of vessel growth impairment and late neo-pulmonary artery stenosis formation [3,7,15,22].

Up to 30% of patients who develop neo-pulmonary artery stenosis after arterial switch operation require additional treatment [6,7,8,9]. There are two treatment options for these patients: surgical and catheter-based interventional treatment. Currently, both in our experience and according to literature, catheter-based interventional treatment is usually the first option, as it is regarded as the safer of the two alternatives [21,23,24,25,26]. Even though, regarded as a safer alternative catheter-based interventional treatment does pose a risk for complications. In their article, Lee et al. reported a 14% incidence of complications (not including the need for subsequent treatment in patients who underwent catheter-based interventional treatment for neo-pulmonary artery stenosis after arterial switch operation [22]. The most common complications are: the need for additional reinterventions, aorto-pulmonic fistulae formation and coronary circulation impairment due to external coronary compression caused by stent placement in the neo-pulmonary artery or its branches. Up to 40% of patients who undergo treatment for neo-pulmonary artery stenosis after arterial switch operation eventually needs additional treatment. The need for subsequent procedures is more common in patients undergoing balloon angioplasty for neo-pulmonary stenosis, though additional procedures may be necessary for patients undergoing redo surgical repair or stent placement [7,9,21,22,27]. Nellis et al., in their paper, suggested stratifying reinterventions based on their anatomical location. The authors argued that if the reinterventions are not analyzed based on location, there could be a report bias—a primary reintervention for main neo-pulmonary artery stenosis, followed by an additional intervention in a neo-pulmonary artery branch could result in falsely deeming the first procedure a failure, while in principal the first intervention may have had a lasting effect for the targeted lesion [9]. In our experience, patients who were treated for neo-pulmonary artery stenosis had reinterventions in the same and new locations. Another two complications: aorto-pulmonic fistulae formation and external coronary compression are regarded as major complications. They require immediate recognition and treatment. Aorto-pulmonic fistulae can form after a simple balloon angioplasty procedure, though it is more common after stent fracture during subsequent stent dilation procedures. Published reports reveal both surgical and catheter-based interventional treatment options were used to successfully treat iatrogenic aorto-pulmonic fistulae [9,22,28,29,30,31,32,33,34]. While external coronary compression was seen in the early days of stent placement in the neo-pulmonary artery or its branches, currently, strict analysis of the anatomy and pre-procedural planning should identify the high-risk cases and help avoid this complication all together [22,35,36]. In our limited experience, we had no aorto-pulmonic fistulae or external coronary compression. This is because not only of the small case volume, but also due to the fact that every single planned procedure is discussed in the multidisciplinary meeting. Our interventional cardiologists have both trans-catheter aortic valve and pulmonary valve replacement programs. Planning for these procedures involve a detailed study of the aortic root, coronary ostia and proximal coronary artery anatomy. This expertise aids in planning catheter-based interventional treatment procedures in patients after arterial switch.

Left-sided lesions are reported less frequently. The most common left-sided lesions after arterial switch operation are neo-aortic root enlargement and neo-aortic valve regurgitation, obstruction of the left ventricle outflow tract, and aortic arch obstruction. While the former two rarely require any treatment at all, left ventricle outflow tract obstruction is usually corrected surgically and aortic arch obstruction (which is usually seen in patients who underwent a repair of hypoplastic aortic arch or aortic coarctation during the arterial switch operation, though it may be caused by a development of new aortic coarctation after arterial switch) may be dealt with surgically or using catheter-based interventional treatment [8,15,21,37,38,39,40]. Stenosis of the native pulmonary valve, bicuspid pulmonary valve and left ventricle outflow tract obstruction prior surgery are the main risk factors for post-switch late obstructive complications, and the main predictors of poor freedom for reinterventions after arterial switch operation [15,21,38,39]. All of our patients treated for left-sided lesions had an aortic arch obstruction. Two patients developed new aortic coarctation after arterial switch operation. One patient underwent surgical resection of aortic coarctation, and the other one underwent balloon angioplasty. Five patients were treated aortic re-coarctation. 2 of them later developed neo-pulmonary stenosis. All patients who were treated with catheter-based interventions underwent at least two balloon angioplasty procedures. One patient underwent stenting of the repeated aortic recoarctation.

## 5. Study Limitations

The study is a retrospective analysis of a single center experience and involves a small number of patients over a long time period. As this study is of retrospective nature, it poses all limitations characteristic to retrospective studies.

## 6. Conclusions

Both pulmonary stenosis and aortic arch obstruction (new aortic coarctations or aortic recoarctations) tend to develop in the first decade after arterial switch operation. Surgical and catheter-based interventional treatment with good results is possible even in a small volume center. Close collaboration of the congenital heart team (congenital cardiac surgeons and interventional cardiologists) in choosing the best treatment option for an individual patient can help to minimize the risk of potential complications.

## Figures and Tables

**Table 1:** Demographic data summary.

	Neo-Pulmonary Stenosis Group	Aortic Arch Obstruction Group
Patient count, *n* (%)	7 (50)	7 (50)
Female:Male, *n*:*n*	2:5	1:6
Transposition form		
TGA-IVS, *n* (%)	2 (28.6)	1 (14.3)
TGA-VSD, *n* (%)	3 (42.8)	1 (14.3)
T-B, *n* (%)	2 (28.6)	5 (71.4) ^a^
Coronary pattern *		
Normal (1LCX2R), *n* (%)	4 (57.1)	2(28.6)
Abnormal, *n* (%)	3 (42.9)	5(71.4)
Additional malformations		
Aortic coarctation, *n* (%)	1 (14.3)	5 (71.4) ^b^
Hypoplastic aortic arch, *n* (%)	-	5 (71.4) ^c^

^a^—Late aortic arch obstruction seems to be more common in patients who underwent arterial switch operation due to Taussig-Bing anomaly, however, the difference between the two groups lack statistical significance (*p*-value is 0.28). ^b^—Late aortic arch obstruction is more common in patients who had concomitant repair of aortic coarctation at the time of arterial switch, though, statistical significance is inconclusive (*p*-value is 0.1). ^c^—Late aortic arch obstruction is significantly more common in patients who underwent concomitant repair of hypoplastic aortic arch at the time of arterial switch operation (*p*-value is less than 0.5). *—Coronary pattern is described in accordance to Leiden convention [11]. Abbreviations: *n*—number; TGA-IVS—transposition of the great arteries with intact ventricular septum; TGA-VSD—transposition of the great arteries with ventricular septal defect; T-B—Taussig-Bing anomaly; 1—first coronary sinus; 2—second coronary sinus; L—left anterior descending artery; CX—circumflex artery; R—right coronary artery.

**Table 2:** Reinterventions and their complications among patients with late arterial switch complications.

	Neo-Pulmonary Stenosis Group	Aortic Arch Obstruction Group
Median time to first reintervention, years (IQR)	2.4 (1.4–2.8)	4 months (2.3 months–1.6 years)
Total number of reinterventions, *n*	17	19
Median number of procedures per patient, *n* (range)	2 (1–5)	2 (1–7)
Balloon angioplasty, *n* (%)	10 (58.8)	13 (68.4)
Stent implantation, *n* (%)	1 (5.9)	2 (10.5)
Surgical repair, *n* (%)	6 (35.3) ^a^	1 (5.3)
Reinterventions in other localization than primary reintervention *, *n* (%)	-	3 (15.8)
Complications, *n* (%)	4 (23.5)	6 (31.5)
Termination of a planned interventional procedure, *n* (%)	1 (25.0)	1 (16.7)
Access site vessel thrombosis or pseudo-aneurysm formation, *n* (%)	-	3 (50.0) ^b^
Target vessel localized dissection, *n* (%)	2 (50.0)	2 (33.3)
Death, related to intervention procedure, *n* (%)	1 (25.0)	-
Complications related to redo cardiac surgery, *n* (%)	-	-

*—Number of reinterventions performed at a different anatomical location than the first reintervention. ^a^—Patients with neo-pulmonary artery stenosis required redo surgical treatment more often than patient with aortic arch obstruction (*p*-value is less than 0.05). ^b^—Among patients who underwent catheter-based interventional treatment, procedure related access vessel complications were more frequent in the aortic arch obstruction group, but the difference lacks statistical significance (*p*-value is 0.2). Abbreviations: IQR—interquartile range; *n*—number.

## Data Availability

The data presented in this study are available on request from the corresponding author.

## Abbreviation

IQR—interquartile range.
